# From Velocity to Acceleration: A Perspective on Age-Related Visual Field Progression in Glaucoma

**DOI:** 10.7759/cureus.85129

**Published:** 2025-05-31

**Authors:** Masaki Tanito, Tomoki Shirakami

**Affiliations:** 1 Department of Ophthalmology, Shimane University Faculty of Medicine, Izumo, JPN

**Keywords:** acceleration, aging, glaucoma, nonlinear modeling, visual field progression

## Abstract

The conventional evaluation of visual field (VF) progression in glaucoma often relies on linear regression to estimate the rate of decline, focusing on velocity (dB/year). However, this approach may underestimate future progression, particularly in elderly patients. This editorial highlights the importance of incorporating acceleration (dB/year²) into clinical assessments of VF loss. Using physiological and pathological models, we demonstrate that VF sensitivity decline is inherently nonlinear with age. A cubic model illustrates age-related sensitivity loss in healthy eyes, while a quadratic model captures the interaction between disease duration and baseline age in glaucomatous eyes. These models emphasize that progression accelerates over time and suggest that individualized treatment intensity should consider not only the current rate of VF decline but also its likely acceleration based on patient age and disease course.

## Editorial

The primary goal of glaucoma therapy is to preserve visual function and the associated quality of life throughout the patient's lifetime [1]. Slowing visual field (VF) loss by lowering intraocular pressure (IOP) remains the most reliable strategy for glaucoma treatment. Therefore, regular assessment of the rate of VF progression is essential when deciding whether the current target IOP is adequate or if additional intervention is required. Large-scale studies have demonstrated that older age is a risk factor for both the onset and progression of ocular hypertension, primary open-angle glaucoma, exfoliation glaucoma, and primary angle closure disease [2-8]. Given that glaucoma is a chronic and progressive disease, these findings suggest that the impact of age on glaucoma progression increases over time.

VF progression is conventionally assessed by determining the rate of change in sensitivity values via linear regression. For example, the Guided Progression Analysis module of the Humphrey Field Analyzer (Carl Zeiss Meditec, Jena, Germany) employs the annual visual field index (VFI) slope (VFI/y) [9], and commercial software such as BeeFiles (BeeLine, Tokyo, Japan) likewise reports the mean deviation (MD) slope (MD/y) [10]. Although these metrics are useful for quantifying current progression speed, they may not fully predict future VF decline. Indeed, age-related vulnerability of neural tissues can lead to accelerated thinning of the retinal nerve fiber layer even at constant IOP levels [11]. Therefore, when evaluating VF progression, it is essential to consider not only the current rate (velocity, dB/y) but also the potential for increasing rate (acceleration, dB/y²). This editorial emphasizes the importance of incorporating the concept of acceleration alongside that of velocity.

Spry and Johnson demonstrated that even in normal eyes, VF sensitivity declines with age in a nonlinear fashion [12]. Their analysis of healthy subjects demonstrated that age-related changes in sensitivity were better captured by a cubic function. The position function (S), which represents the VF sensitivity in decibels at age x in years, is defined as $\text{S}\left( \text{x} \right)=-8.54&times;10^{⁻⁶}&times;\text{x}^{&sup3;}+30.5$.

In their original work, only this position function was presented [12]. For the purpose of this editorial, we further derived the first and second derivatives of the position function to represent the rate of change and acceleration of VF loss with age. The velocity function (S/y), describing the yearly rate of VF sensitivity decline, is given by $\text{S&rsquo;}\left( \text{x} \right)= -2.562 &times; 10^{⁻⁵}&times;\text{x}^{&sup2;}$. The acceleration function (S/y²), indicating the age-related acceleration of sensitivity loss, is $\text{S&rsquo;&rsquo;}\left( \text{x} \right)=-5.124&times;10^{⁻⁵}&times;\text{x}$. 

**Figure 1:**
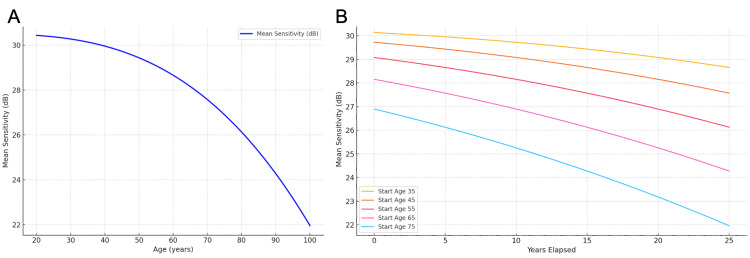
Age-related decline in VF sensitivity in normal eyes, based on Spry and Johnson's cubic model. (A) Projected lifelong trajectory of VF sensitivity for a baseline age of 20 years. (B) Projected 25-year trajectories of VF sensitivity for baseline ages of 35, 45, 55, 65, and 75 years. VF: visual field Reference: [12]

These derived expressions demonstrate that even in the absence of disease, the decline in sensitivity is not constant but accelerates with age (Figure 1A, 1B). This physiologic baseline serves as a foundation for comparison with glaucomatous damage.

In glaucomatous eyes, Shirakami et al. [13] proposed a quadratic model to describe the progression of VF loss over time, incorporating time since diagnosis (t) and baseline age (b). The position function (MD), also denoted as y(t), is given by $\text{y}\left(\text{t}\right)=-0.004&times;\text{t}^{2}+\left(-0.004&times;\text{b}+0.02\right)&times;\text{t}+\text{C&rsquo;}$, where C’ is a constant representing the baseline MD. The velocity function (MD/y), or y’(t), representing the yearly rate of MD change, is $\text{y&rsquo;}\left( \text{t} \right)= -0.008 &times;\text{t}- 0.004 &times;\text{b}+ 0.02$. The acceleration function (MD/y²), or y”(t), which describes the constant rate of acceleration in MD loss, is $\text{y&rdquo;}\left(\text{t}\right)= -0.008$.

**Figure 2:**
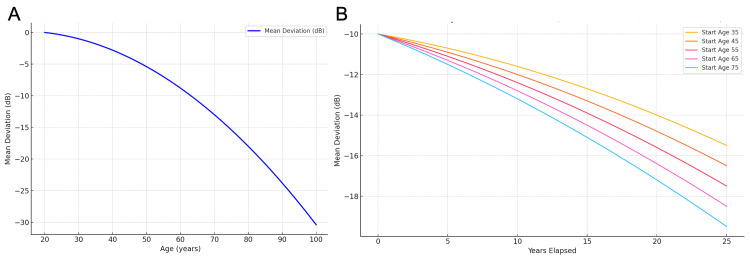
Age-related VF decline in glaucomatous eyes derived from the Shirakami et al. quadratic model. (A) Projected lifelong trajectories of VF sensitivity (starting MD = 0 dB; baseline age = 20 years). (B) Projected 25-year trajectories of VF MD in glaucomatous eyes starting from −10 dB for baseline ages of 35, 45, 55, 65, and 75 years. VF: visual field; MD: mean deviation Reference: [13]

In this model, the decline in MD is not only nonlinear but also dependent on the patient's age at diagnosis. Older patients exhibit a higher progression rate, highlighting the compounding effects of aging and disease duration (Figure 2A, 2B).

These VF progression models [12,13] demonstrate that relying solely on linear slopes risks underestimating true progression, especially in elderly patients. In the clinical setting, incorporating the concept of potential acceleration in disease progression may influence treatment strategies, patient counselling, and clinical outcomes, particularly when considering current progression rate and patient life expectancy. It is desirable that future software for VF assessment include metrics related to acceleration.
